# Change in treatment preferences in pediatric diaphyseal forearm fractures: a Danish nationwide register study of 36,244 fractures between 1997 and 2016 (Hansen et al. Acta Orthop 2023; 94: 32–7)

**DOI:** 10.2340/17453674.2024.40813

**Published:** 2024-06-10

**Authors:** Ole Rahbek, Søren Kold, Hans-Christen Husum

**Affiliations:** Interdisciplinary Orthopaedics, Aalborg University Hospital, Denmark

*Sir*,—We have with great interest read the study “Change in treatment preferences in pediatric diaphyseal forearm fractures: a Danish nationwide register study of 36,244 fractures between 1997 and 2016” by Hansen et al. 2023 [1]. We would like to comment on the validity of the register data used.

The primary objective of this study was to examine the treatment patterns for diaphyseal forearm fractures in Danish pediatric patients over a span of 2 decades, utilizing registry data from the Danish National Patient Register (DNPR). However, we express reservations regarding the validity of the data utilized and, consequently, the robustness of the findings presented in this paper.

We commend the authors for addressing this concern by referencing previous work stating, “The positive predictive value of a correct primary diagnosis in orthopedic surgery is 83%” and acknowledging that “the specific rate has not been investigated in diagnostics of fractures.” However, this raises significant doubts concerning the foundation upon which the data analysis rests.

The reference provided [2] evaluates the validity of diagnostic and procedure codes in DNPR, deriving the 83% positive predictive value from analyses of periprosthetic hip joint infection, traumatic hip dislocations, and general “orthopedic surgery.” Notably, since 2015, 3 studies have examined the validity of fracture diagnoses in the DNPR, specifically focusing on hip, ankle, and humeral fractures [3-5], albeit none involving pediatric populations.

These discrepancies lead us to question the susceptibility of the paper to information bias. Furthermore, the substantial proportion (30%) of fractures categorized as diaphyseal, which were isolated radius fractures, raises additional concerns regarding potential bias inherent in the dataset, particularly to those with expertise in the field.

Additionally, the authors’ rationale for including Kirschner wires in the treatment cohorts, under the assertion that they are commonly used as an alternative to intramedullary nails (IMN) for pediatric diaphyseal forearm fractures, warrants scrutiny. While this practice may hold true for younger children, the indication that nearly 10% of diaphyseal forearm fractures in older children (aged 8–15 years) were treated with K-wires appears dubious and could signify either misclassification of distal forearm fractures as diaphyseal fractures or misclassification of the operative procedure within the source data.

In our view, any study utilizing registry data should prioritize ensuring the accuracy and validity of the data employed, through either comprehensive literature review or direct validation efforts. Ideally, the data should be validated against the gold standard (i.e., the actual radiograph or medical record) before use to ensure the data quality. We hope that the authors can provide information on the validity of the diagnosis codes and procedure codes used for this specific anatomical location of fractures in children within the DNPR.

We believe this information is needed for a rigorous approach to data validation in future publications by *Acta Orthopaedica* to uphold the integrity of research findings.

**Ole Rahbek, Søren Kold, and Hans-Christen Husum**

*Interdisciplinary Orthopaedics, Aalborg University Hospital, Denmark*

*Correspondence: h.husum@rn.dk*
